# Impact of fetal spine alignment according to maternal lateralization during early labor on maternal comfort and birth outcomes: A prospective cohort study in Kelantan, Malaysia

**DOI:** 10.18332/ejm/191737

**Published:** 2024-09-02

**Authors:** Nafila Abdul Rahman, Erinna Mohamad Zon, Engku Husna Engku Ismail, Nik Ahmad Nik Abdullah, Wan Mohd Zahiruddin Wan Mohammad, Rahimah Abdul Rahim, Nik Ahmad Zuky Nik Lah

**Affiliations:** 1 Department of Obstetrics and Gynecology, School of Medical Sciences, Universiti Sains Malaysia, Kelantan, Malaysia; 2 Department of Obstetrics and Gynecology, Hospital Raja Perempuan Zainab II, Kelantan, Malaysia; 3 Hospital Universiti Sains Malaysia, Kelantan, Malaysia; 4 Department of Community Medicine, School of Medical Sciences, Universiti Sains Malaysia, Kelantan, Malaysia

**Keywords:** fetal spine position, maternal comfort, labor, maternal-fetal position

## Abstract

**INTRODUCTION:**

Maternal positioning during labor significantly influences maternal comfort. This study aims to identify the preferred maternal lateral position during the latent phase and examine the impact of alignment between maternal lateralization and fetal spine positioning during the active phase of the first stage of labor on maternal comfort.

**METHODS:**

Pregnant women in the first stage of labor beyond 37 weeks of gestation were recruited over six months from March to August 2020 for this prospective cohort study at Hospital Raja Perempuan Zainab II, Kota Bharu, Kelantan, Malaysia. Eligible individuals were randomly allocated to align with the fetal spine (n=180) or oppose it (n=180). Fetal spine positions were confirmed via transabdominal ultrasound. Maternal mean comfort scores were assessed using the established Maternal Comfort Assessment Tool. Statistical analysis was performed using IBM SPSS version 27, with a p<0.05 considered significant.

**RESULTS:**

There was a significant association between the preferred maternal position during the latent phase and concordance with the same maternal lateralization-fetal spine alignment (p<0.001). Higher mean comfort scores were observed when the maternal lateral position matched the fetal spine alignment during the active phase of labor. There was a significant association of normal CTG tracings when the maternal position was aligned with the fetal spine (p<0.001).

**CONCLUSIONS:**

Parturients preferred lying in alignment with the fetal spine lateralization during the latent phase. This position also offers increased comfort during the active phase of labor. It highlights the importance of considering maternal–fetal alignment as a critical factor in intrapartum care.

## INTRODUCTION

Spontaneous, uncomplicated vaginal delivery involves dynamic processes influenced by the position, movements, descent, and delivery of the fetus. It is a painful experience due to intense uterine contractions and can be tiring and frightening for the laboring mother^1^. Providing maternal comfort, especially through alleviating labor pain and anxiety, is known to influence the labor process and has been extensively studied^2^. Various techniques and methods have been researched to reduce labor pain, including hypnosis^3^, biofeedback^4^, immersion in water^5^, intracutaneous or subcutaneous sterile water injection^6^, aromatherapy^7^, relaxation techniques^2^, acupuncture^8^, massage, and reflexology^9^. These methods work by interrupting the transmission of pain signals, limiting the capacity to pay attention to pain, stimulating the release of endorphins, or helping to diminish pain-exacerbating thoughts^10,11^.

Additionally, anesthesia and analgesics such as transcutaneous electrical nerve stimulation (TENS)^12^, inhaled analgesia^13^, opioid and non-opioid drugs^14^, local anesthetic nerve blocks^15^ and epidurals^16,17^, play crucial roles in labor pain management. Labor companions also significantly influence maternal comfort^18^. Moreover, various maternal positions during labor have been identified to promote maternal comfort and successful vaginal delivery with good perinatal outcomes^19^.

Little is known about the fetal comfort position *in utero*. Fetus activities and positional changes *in utero* can be either physiologically or pathologically related^20^. A healthy fetus typically remains calm without cord compression *in utero*. Persistent cord compression, such as with a tight nuchal cord, may initially cause the fetus to struggle for oxygen. This struggle is perceived antenatally as an increase in fetal activity, which can also lead to maternal discomfort. If cord compression persists and is not resolved, fetal hypoxia can ensue, resulting in reduced movement and potentially leading to intra-uterine death^20^. Fetal position is primarily influenced by comfort *in utero*, possibly associated with certain maternal positions that promote normal fetal movement and activities or reduce cord compression. Laboring mothers are often advised to lie in the left lateral position to enhance comfort, as this position alleviates inferior vena cava (IVC) compression caused by the contracting gravid uterus. By combining considerations of both fetal and maternal comfort, it may be possible to increase the comfort level of laboring mothers and promote uneventful vaginal deliveries.

During the third trimester, the maternal supine position significantly reduces cardiac output due to compression of the IVC by the gravid uterus, potentially leading to supine hypotensive syndrome^21,22^. Interestingly, sleeping in a supine position during this period is associated with a 2.6-fold increase in late stillbirth^23^. However, left or right lateral positioning during sleep appears equally safe^23^, with 50.6% of pregnant mothers preferring the left lateral position and 43.8% opting for the right lateral position^21^. These findings suggest the safety of maternal lateralization during the antenatal period is likely influenced by individual comfort, a factor not thoroughly explored previously.

A prior study proposed a theory elucidating the relationship between maternal–fetal occiput positioning and the center of gravity of the fetus *in utero*^21^. A fetus with a left occiput position predominantly places its body on the mother’s left side. When the mother assumes a left lateral position, the center of gravity of the fetus shifts to the mother’s left side, encouraging the fetus to settle and maintain its position *in utero*^21^.

In numerous studies, ultrasound is a remarkable tool for determining fetal occiput and spine position during the first and second stages of labor^24,25^. Knowledge about the preferred lateral position of full-term mothers antenatally has not been taken seriously, as it is commonly believed that the only safe position is the left lateral position. However, many women find comfort in lying on the right lateral position^21^. What could be the contributing factors? Could we adopt the right lateral position during labor? Is it safe to both the mother and fetus?

Based on the concordance between the fetal spine and respective maternal lateralization positions, we hypothesize that fetal spine position may influence maternal comfort both antenatally and during labor. Thus, this study aims to elucidate the preferred maternal lateralization concerning fetal spine position in early labor and investigate the impact of maternal lateralization-fetal spine concordance on maternal comfort during the first stage of labor. Additionally, its association with the duration of labor, mode of delivery, requirement for analgesia, and Apgar scores at birth was also investigated.

## METHODS

### Study design and setting

This prospective cohort study engaged pregnant women who met rigorous inclusion criteria and attended Hospital Raja Perempuan Zainab II (HRPZII) in Kelantan, Malaysia, for delivery between March 2020 and August 2020. Recruitment occurred over 6 months from 1 March to 31 August 2020.

Eligible women from 28 weeks of gestational age attending antenatal clinic appointments at HRPZII were approached for recruitment. They were provided with detailed patient information sheets explaining the study’s nature, and informed consent was obtained before participation. Participants recruited during antenatal follow-up continued their follow-up visits in the antenatal clinic until their admission for delivery. The recruitment process adhered to ethical standards, ensuring voluntary consent after the participants fully understood the study details. Participants retained the right to withdraw their consent at any time.

Additionally, all pregnant women at term pregnancy admitted in early labor were invited to participate in the study upon admission at the Patient Assessment Centre (PAC). Baseline demographic data were recorded during admission, including maternal parity, age, height, weight, and body mass index (BMI, kg/m^2^). The preferred maternal position was carefully evaluated at this time.

### Participants

Eligible women who met the inclusion were selected, and a sample number was assigned to each subject’s datasheet to maintain confidentiality. The inclusion criteria included women aged ≥18 years, carrying a live, normal singleton fetus in cephalic presentation, without underlying maternal medical co-morbidities, in the latent phase of labor (cervical dilatation <4 cm), with a maternal BMI falling between 18.5–35 kg/m², and an estimated fetal weight (EFW) between 2.5–3.5 kg. The specified EFW range aimed to standardize the sample, with a precision error of ±500 g, while macrosomic fetuses with an EFW of 4.0 kg were excluded.

The exclusion criteria included prior uterine surgeries, medical or obstetric complications (such as preeclampsia, cardiovascular disorders, any form of diabetes, induction of labor, premature rupture of membrane, epidural anesthesia), fetuses with a nuchal cord observed on admission ultrasound, and cases of oligohydramnios (AFI <5 cm) or polyhydramnios (AFI >25 cm) or distress based on CTG monitoring. These exclusions were implemented considering their potential impacts on labor progression and maternal or fetal outcomes.

The sample size was calculated using kappa statistics. The primary outcome, based on previous data by Matsuo et al.^21^ on the relationship between maternal positioning in late pregnancy and fetal positioning *in utero*, was used to calculate the sample size: 50.6% probability of fetal left-back position; 51.7% probability of women preferring left lateral position; power=0.95; alpha=0.05; -k1=0.75; and k0=0.65. Using the incidence rate ratio (IRR) package, the calculated sample size was 327. Assuming a dropout rate of 10%, the total sample size was determined to be 360.

Maternal preference for the position at the beginning of labor, either lying in the same lateralization as the fetal spine or opposite to the fetal spine, was assessed during admission to the labor room. An ultrasound examination was conducted to reassess the fetal occiput and spine positions. An ultrasound examination form recorded fetal spine and occiput position data.

Then, they were allocated the next available numbers in a concealed sequence of computer-generated randomization to determine the maternal position to be adopted during labor. Participants were randomly assigned to one of two maternal position groups: the same lateralization as the fetal spine (n=180) and opposite to the fetal spine (n=180).

Depending on their assigned position, participants were asked to maintain the selected position throughout the labor process. If the labor duration exceeded 4 hours, adherence to the assigned position was required for at least 80% of the labor duration. Labor was monitored using a partogram, and fetal heart rate and cardiotocography were continuously monitored via obs-central.

### Variables

The primary study variable was the preferred maternal lateral position during the latent phase and the impact of alignment between maternal lateralization and fetal spine positioning during the active phase of the first stage of labor on maternal comfort. Maternal comfort was assessed using the Maternal Comfort Assessment Tool developed by Chrzanowski and Young^26^. Both primary outcome (maternal comfort level) and secondary outcome measures (labor duration, mode of delivery, pain relief, cardiotocography (CTG) reactivity, and fetal condition at birth based on Apgar score) were recorded on patient proforma. Additional data collected included maternal age, gestational age at delivery, parity, birthweight, body mass index (BMI), augmentation requirement, baby’s sex, and resuscitation requirement for the newborn. The presence of specific birth companions or doulas was not applicable in the government hospital setting.

**Figure 1:**
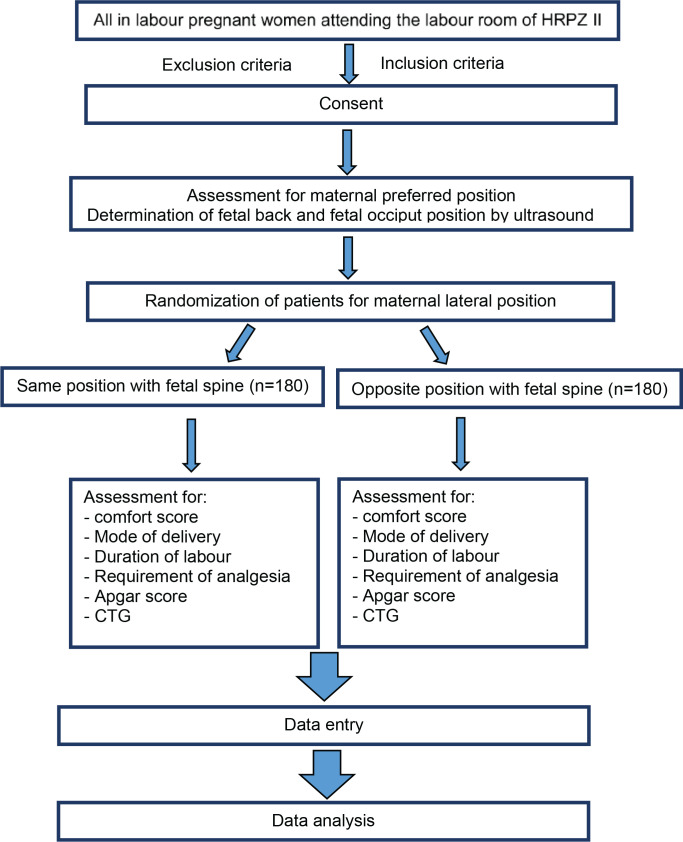
Flow diagram of patient recruitment of the study

### Measurement

A designated medical officer assigned to the study conducted a transabdominal ultrasound scan to determine the fetal head (occiput) and spine positions. The occiput position was identified by placing the ultrasound transducer suprapubically in the transverse plane to visualize the fetal orbits and midline cerebral echo, especially the paired thalami in the transverse view of the fetal head^27^. Subsequently, the transducer was positioned on the maternal abdomen to obtain a transverse view of the fetal trunk at the upper fetal abdomen level or the heart’s four-chamber view. The transducer was then rotated longitudinally to obtain a sagittal plane view of the fetal spine, facilitating the determination of the fetal spine position^28^. Intrapartum ultrasonography showed a sensitivity of 100%, specificity of 98%, positive predictive value (PPV) of 85%, and negative predictive value (NPV) of 100% in determining fetal spine position^24^.

Comfort scores were evaluated and recorded by treating doctors every two hours during the active phase of labor. The average was calculated to determine a mean comfort score for each woman. Higher scores indicated greater comfort during the delivery process, with a maximum score of 14. This tool was divided into seven subcategories (focus of attention, eye contact during contraction, breathing pattern during contraction, vocal behavior with contraction, muscle tension with contraction, activity during contraction, and verbalization), and each subcategory was scored from 0 to 2^26^.

### Statistical analysis

Statistical analysis was performed utilizing SPSS (IBM Company, Chicago, IL, USA) version 27. Continuous data were expressed as mean ± standard deviation (SD), whereas categorical data were depicted as frequencies and percentages. Missing data were checked, and any imputation and sensitivity analyses were conducted accordingly. The associations among maternal lateral position, fetal spine concordance, preferred maternal position, maternal comfort score, delivery specifics, and neonatal outcomes were evaluated using independent t-tests for continuous data and Fischer’s exact or chi-squared test for categorical data. Concordance was assessed using Cohen’s kappa statistic, with statistical significance at p<0.05.

### Ethics

Ethical approval was obtained from the Universiti Sains Malaysia, Human Research Ethics Committee (JEPeM Code: USM/JEPeM/19120931, dated 12 March 2020) and the Medical Research and Ethics Committee (MREC), Ministry of Health Malaysia (MOH) (Code: NMRR-19-3698-52089 (IIR) dated 23 February 2020). Informed written consent was diligently obtained from all participants.

## RESULTS

Over a span of six months, a total of 360 participants were enrolled in the study. The mean age was 27.9 years (SD=5.5), and the mean gestational age at the onset of labor was 38.99 weeks (SD=0.99). Nulliparous women comprised 41.9% of the cohort. Regarding BMI classifications, 38.6% were categorized as overweight (25–29.9 kg/m^2^), 28.9% as obese (30–35 kg/m^2^), and 32.5% fell within the normal range (18.5–24.9 kg/m^2^) (Supplementary file Table 1).

Among the 360 participants, 180 displayed concordant maternal-fetal spine positions, while 180 exhibited discordance during labor. Approximately 30% required labor augmentation. All participants delivered live fetuses, with a mean birth weight of 3134.5 g (SD=337.3), and the majority of the neonates were male (55.8%). Neonatal resuscitation was unnecessary for 67.5% of neonates, with only one requiring intubation. Among all deliveries, 30.8% of neonates necessitated suction, while 0.6% required facial oxygen and positive pressure ventilation (Supplementary file Table 1).

### Correlation between maternal positioning preferences and fetal spine alignment assessed via ultrasound during early labor

Table 1 indicates a significant association between fetal spine and maternal preferred positions (p<0.001). Moreover, Table 2 demonstrates a high agreement between the fetal occiput position and its corresponding spine alignment, as evidenced by Cohen’s kappa of 0.96^29^.

**Table 1:** The association between fetal spine position and maternal preferred position among pregnant women at the Hospital Raja Perempuan Zainab II (HRPZ II), Kelantan, Malaysia, March to August 2020 (N=360)

*Variables*	*Fetal spine position*		p*
	*Left n (%)*	*Right n (%)*	
**Maternal preferred position**			
Left	147 (66.2)	51 (37.0)	<0.001
Right	75 (33.8)	87 (63.0)	

* Pearson chi-squared test.

**Table 2:** The agreement between fetal spine position and the occiput position among pregnant women at the Hospital Raja Perempuan Zainab II (HRPZ II), Kelantan, Malaysia, March to August 2020 (N=360)

*Variables*	*Fetal spine position*		*Total (N=360) n*	*Cohen’s kappa*
	*Left (N=222) n*	*Right (N=138) n*		
**Fetal occiput position**				
Left	217	2	219	0.959
Right	5	136	141	

### Maternal comfort score

Table 3 reveals that the mean comfort score was significantly higher when the maternal lateralization matched the fetal spine position while Table 4 indicates no statistically significant difference in mean labor duration among various maternal position groups. Table 5 indicates no significant association between the mode of delivery and the need for analgesia across the maternal position groups. However, it highlights a higher analgesia utilization in the discordant lateralization group. Additionally, most participants (75%) exhibited a normal CTG with a baseline heart rate between 110–160 bpm and baseline variability between 5–25 bpm, along with accelerations and no deceleration^30^. Notably, a significant association (p<0.001) was observed between different maternal position groups and CTG, with the same position associated with a higher incidence of normal CTG. Over 95% of neonates achieved a favorable Apgar score (8–10) at 1 min and at 5 min post birth. However, no significant association was found between the maternal position groups and the Apgar scores at 1 min and at 5 min post birth (p=0.139 and p=0.623, respectively).

**Table 3:** Comparison comfort score between various maternal positions among pregnant women at the Hospital Raja Perempuan Zainab II (HRPZ II), Kelantan, Malaysia, March to August 2020 (N=360)

*Variable*	*Maternal–fetal spine concordance*		*Mean difference (95% CI)*	*t-statistic (df)*	p*
	*Same Mean (SD)*	*Opposite Mean (SD)*			
Comfort score	11.5 (3.94)	10.5 (3.68)	0.95 (0.16–1.74)	2.37 (358)	0.013

* Independent t-test.

**Table 4:** Comparison duration of labor between various maternal positions among pregnant women at the Hospital Raja Perempuan Zainab II (HRPZ II), Kelantan, Malaysia, March to August 2020 (N=360)

*Stage of labor*	*Maternal–fetal spine concordance*		*Mean difference (95% CI)*	*t-statistic (df)*	p*
	*Same Minutes mean (SD)*	*Opposite Minutes mean (SD)*			
First	149 (141)	152 (129)	-3.97 (-32.05–24.10)	-0.28 (357)	0.781
Second	7 (5)	8 (7)	-1.18 (-2.43–0.06)	-1.87 (357)	0.063

* Independent t-test was applied since sample size (n) in both groups is >30.

**Table 5:** Comparison mode of delivery, analgesia requirement, CTG normal and Apgar score between different groups of maternal position among pregnant women at the Hospital Raja Perempuan Zainab II (HRPZ II), Kelantan, Malaysia, March to August 2020 (N=360)

*Variables*	*Maternal–fetal spine concordance*		*p*
	*Same n (%)*	*Opposite n (%)*	
**Mode of delivery**			0.261^b^
SVD	155 (86.1)	159 (88.3)	
VAD	6 (3.3)	10 (5.6)	
FD	2 (1.1)	0 (0)	
CS	17 (9.5)	11 (6.1)	
**Mode of delivery**			0.528^a^
SVD	155 (86.1)	159 (88.3)	
Others	25 (13.9)	21 (11.7)	
**Analgesia**			0.675^b^
No	117 (65.0)	113 (62.8)	
Nalbuphine	60 (33.3)	64 (35.6)	
Nitrous oxide	3 (1.7)	3 (0.8)	
**CTG**			<0.001^b^
Normal	153 (85.0)	117 (65.0)	
Suspicious	21 (11.7)	60 (33.3)	
Pathological	6 (3.3)	3 (1.7)	
**Apgar score at 1 min**			0.139^b^
0–3	0 (0)	1 (0.6)	
4–7	3 (1.7)	8 (4.4)	
8–10	177 (98.3)	171 (95.0)	
**Apgar score at 5 min**			0.623^b^
4–7	1 (0.6)	3 (1.7)	
8–10	179 (99.4)	177 (98.3)	

a Pearson chi-squared.

b Fisher’s exact test.

## DISCUSSION

### Principal findings

The study revealed a significant association between the fetal spine position and the mother’s preferred lying position during the latent phase of labor. Our study observed that maternal lateralization aligned with the fetal spine position resulted in a higher mean comfort score for the mother during the active phase of labor. Additionally, a significant association was observed during labor between maternal–fetal spine alignment and normal fetal heart rate pattern on cardiotocography (CTG). However, no significant association was found between maternal–fetal back positioning and the mode of delivery, with similar rates of spontaneous vaginal delivery across different positions. The study also identified no correlation between maternal–fetal alignment and labor outcomes such as pain relief, labor duration, the need for interventions (including operative vaginal delivery or cesarean section), or Apgar scores of the newborn.

Statistical analyses revealed a significant association between the fetal spine and the mother’s preferred position. The most common combination was a left-sided fetal spine with the left lateral position, with more than half of women with a left-sided fetal spine preferring this position. Similarly, those with a right-sided fetal spine tended to prefer the right lateral position. This suggests that the fetal spine position influences maternal comfort, as women favored lying on the same side as the fetal spine. A previous study proposed a theory to explain the relationship between maternal–fetal occiput positioning *in utero* and the center of gravity of the fetus^21^. The theory suggests that a fetus with a left occiput position predominantly occupies the mother’s left side. Consequently, when the mother assumes a left lateral position, the center of gravity of the fetus in the left spine position shifts further to the mother’s left side, potentially encouraging the fetus to settle into and maintain this position *in utero*. However, a study investigating maternal sleeping position during the latter half of pregnancy found no statistical correlation between maternal lateralization and fetal occiput positioning *in utero*^23^.

Concordance between the fetal spine and occiput positions during the active phase of the first stage of labor was high, indicating strong agreement^29^. Only 2% of participants showed discordance between the occiput and spine positions during the first stage of labor. This occurrence is likely attributed to the engagement of the head into the pelvis primarily in a transverse position, thereby causing the fetal spine to align accordingly with reduced movement. Our study supports the hypothesis that fetal spine positioning affects maternal comfort, as a maternal preference for lying on the same side as the fetal spine. This increased comfort is likely due to factors such as a calm fetus, adequate amniotic fluid volume, and the absence of nuchal cord compression. Cord compression may cause fetal struggling and, subsequently, maternal discomfort. A calm fetus with an occipital transverse position and a concordance fetal spine position is more likely to facilitate occiput flexion and fixation within the pelvic cavity, thereby reducing occiput malposition.

We observed that the fetal spine and occiput were predominantly aligned on the same side, with a higher prevalence of the left fetal spine than the right fetal spine position. The concordance analysis indicated a solid agreement where the fetal occiput position exhibited an almost equal chance likelihood of presentation with the corresponding fetal spine position during early labor^29^. Notably, in the current study, discordance between the occiput and spine position occurred in only 2% of participants during the first stage of labor. This occurrence is likely attributed to the engagement of the head into the pelvis primarily in a transverse position, thereby causing the fetal spine to align accordingly with reduced movement.

Previous studies have primarily focused on the fetal spine and occiput positions without examining the maternal lying positions before induction of labor or during labor and their impact on labor outcomes^31-33^. There has been a notable lack of information regarding maternal positioning, which could influence the rotation of the fetal presenting part, particularly in cephalic presentations. Gizzo et al.^33^ concluded that evaluating the fetal spine position during the first stage of labor was highly accurate in predicting the fetal occiput position at birth. Thus, if the fetal position could be maintained lateral with maternal lateralization aligned with the fetal spine, the normal vaginal delivery could be successful.

The maternal position during the first stage of labor involves different upright positions (including walking, sitting, standing and kneeling) and recumbent positions (supine, semi-recumbent and lateral) as practiced and directed by midwives or birth attendants^34^. However, such positions may not always be comfortable for both the mother and the fetus. While certain maternal positions can promote fetal comfort and encourage normal occiput rotation to the anterior, they can also result in fetal discomfort and vigorous movements, subsequently hypoxia and becoming muscular hypotonia, particularly in cases of undiagnosed nuchal cord compression^20^. This may lead to malrotation of the fetal occiput, poor progress, or acute fetal distress, as indicated by CTG monitoring^20^.

Our study observed that maternal lateralization concordant with the fetal spine position resulted in a higher mean comfort score for the mother. This could be attributed to factors such as adequate amniotic fluid levels and the absence of nuchal cord compression. A calm and healthy fetus is more likely to facilitate occiput flexion and fixation within the pelvic cavity, thereby reducing occiput malposition. This effect is further enhanced when the fetal spine is positioned laterally.

The study’s results indicated no noteworthy link between various maternal–fetal back positions and the mode of delivery. Both groups exhibited a similar rate of spontaneous vaginal delivery (SVD), with no statistically significant difference observed. Moreover, membership in the same lateralization group did not correlate with a decrease in the requirement for pain relief, labor duration, or intervention use. Neither operative vaginal delivery nor cesarean section demonstrated any association with fetal spine positions, according to the study’s findings.

### Clinical implications

This outcome bolsters our hypothesis that the positioning of the fetal spine affects maternal comfort, suggesting that the mother’s favored lateralization correlates with the fetal spine being positioned on the same side. An important finding emerged regarding the association between maternal positions and CTG. The majority of participants exhibited a normal CTG in both groups. A significant correlation was identified between maternal–fetal spine alignment and a normal fetal heart rate pattern on CTG. Notably, women lying on the opposite side from the fetal back displayed a notably higher occurrence of suspicious CTG patterns, with a frequency three times greater compared to those in the same side-lying position. Further investigation is warranted to elucidate this phenomenon. Could discordance in fetal spine position and maternal lateralization contribute to fetal discomfort *in utero*, potentially leading to increased fetal movements and a heightened risk of cord compression? Alternatively, could this position promote hyperextension of the fetus’s neck? Previous studies, however, have primarily compared the effect of fetal heart rate tracing with different maternal positions rather than directly analyzing maternal–fetal positioning during labor^35^.

### Research implications

The maternal position adopted during normal labor may be crucial for alleviating labor pain and assisting in the fetal head engagement in the transverse position. This facilitates descent with anterior head rotation within the pelvic cavity, reducing the risk of fetal occiput malrotation, and is associated with favorable labor outcomes^34^. We advocate for adopting maternal lateralization based on the side of the fetal spine during the intrapartum period. The attending doctor should evaluate the fetal spine position and fetal head engagement through abdomen palpation, confirm the alignment of the fetal spine, and rule out the presence of a nuchal cord via ultrasound examination before directing the mother to the appropriate lateral position.

This approach suggests a promising avenue for further research, particularly in delineating the optimal maternal labor position in complex scenarios, such as cases involving fetuses with a nuchal cord during labor. Investigating maternal position determined by fetal spine lateralization could significantly improve maternal comfort and labor outcomes, highlighting its potential for widespread clinical application.

### Strengths and limitations

The study’s findings can be applied in settings where a mother is required to be in a recumbent position. Most hospitals and birth centers worldwide adopt policies requiring a recumbent position for mothers in labor. Healthcare providers in these different labor room settings could adopt similar practices, aligning maternal lateralization with fetal spine positioning to enhance maternal comfort and potentially improve fetal outcomes.

In centers with limited ultrasound resources and trained ultrasonography personnel, the diagnosis of the nuchal cord may be missed, which can be associated with cord compression and subsequent fetal distress if the mother adopts the lateralization to the fetal spine during labor. Therefore, maternal lateralization aligned with the fetal spine is recommended only for fetuses without a nuchal cord or with a single, loose nuchal cord and normal CTG monitoring. Further research is needed to explore the applicability of this practice in the presence of nuchal cord.

A limitation of the study is its reliance on comfort scoring, which is inherently subjective and influenced by individual pain perception. Women exhibit varying pain thresholds, making it challenging to standardize comfort scoring across parturients. Additionally, the administration of analgesia and labor augmentation could further impact individual comfort scores, potentially introducing additional variability into the results. Despite these limitations, the study highlights the potential benefits of maternal positioning based on fetal spine lateralization, suggesting a promising area for further research, particularly in complex labor scenarios.

We acknowledge the limitations of not controlling for confounding variables when determining the relationship between maternal factors, such as amniotic fluid volume and maternal pelvic shape, and the outcome variables. Future studies should employ statistical methods, such as regression models, to adjust for these potential confounding effects.

## CONCLUSIONS

This prospective study shows a strong association between the fetal spine, occiput positions, and the mother’s preferred lateralization. The alignment of maternal and fetal positions, especially when both are oriented laterally in the same direction, significantly enhances maternal comfort during labor. While no definitive associations were found with traditional labor parameters, such as duration, mode of delivery, analgesia requirement, and Apgar scores, a noteworthy connection was observed between maternal–fetal alignment and a normal cardiotocography (CTG) pattern. Underscoring a potential link between maternal–fetal alignment and fetal well-being.

This nuanced understanding contributes valuable insights into optimizing maternal positioning to enhance comfort and improve fetal outcomes during labor, emphasizing the importance of maternal–fetal alignment in intrapartum care. These findings open avenues for future research, encouraging further exploration of maternal–fetal positioning for improved management in diverse clinical scenarios. Such research could lead to advancements in intrapartum care and better outcomes for both mothers and babies.

## Supplementary Material



## Data Availability

The datasets analyzed for the study are available with permission from the corresponding author upon on reasonable request.
