# Supplementary material for: Impact of fetal spine alignment according to maternal lateralization during early labor on maternal comfort and birth outcomes: A prospective cohort study in Kelantan, Malaysia
Source: Eur J Midwifery. 2024 Sep 2;8:10.18332/ejm/191737. doi: 10.18332/ejm/191737 (PMC11367680; doi:10.18332/ejm/191737)
Supplement: Supplementary file 1 [file EJM-8-49-s1.pdf]

Table 1: Maternal and neonatal characteristics according to maternal-fetal spine concordance among 360 pregnant women at the Hospital Raja Perempuan Zainab II (HRPZ II), Kelantan, Malaysia, March to August 2020 (N=360)

**Variables**	**Maternal-fetal spine concordance**		
	**Total (n= 360)**	**Same (n=180)**	**Opposite (n=180)**
	**Mean (SD)**	**Mean (SD)**	**Mean (SD)**
Age (years)	27.9 (5.5)	27.9 (4.9)	27.9 (6.0)
Gestational age (weeks)	38.9 (1.0)	38.9 (1.0)	39.1 (1.0)
Birthweight (gm)	3135 (338)	3139 (302)	3130 (371)
		n(%)	n(%)
Parity			
Nulliparous		74 (41.1)	77 (42.8)
Parous		106 (58.9)	103 (57.2)
BMI (kg/m2) category			
Normal (18.5-24.9)		60 (33.3)	57 (31.7)
Overweight (25-29.9)		71 (39.4)	68 (37.8)
Obese (30-35)		49 (27.2)	55 (30.5)
Augmentation			
Yes		51 (28.3)	55 (30.6)

No	129 (71.7)	125 (69.4)
Baby sex		
Boy	95 (52.8)	106 (58.9)
Girl	85 (47.2)	74 (41.1)
Resuscitation		
None	116 (64.4)	128 (71.1)
Suction only	63 (35.0)	48 (26.7)
Facial oxygen	0 (0.0)	2 (1.1)
PPV	0 (0.0)	2 (1.1)
Intubation and drugs	1 (0.6)	0 (0.0)

BMI = Body Mass Index

PPV = Positive Pressure Ventilation

© 2024 Nik Lah N.A.Z. et al.
